# *In-vitro *antimycobacterial drug susceptibility testing of non-tubercular mycobacteria by tetrazolium microplate assay

**DOI:** 10.1186/1476-0711-7-15

**Published:** 2008-07-11

**Authors:** Manimuthu Mani Sankar, Krishnamoorthy Gopinath, Roopak Singla, Sarman Singh

**Affiliations:** 1 Clinical Microbiology Division, Department of Laboratory Medicine, All India Institute of Medical Sciences, New Delhi, India; 2 Division of Respiratory Medicine, LRS Institute of Tuberculosis and Lung Diseases, New Delhi, India

## Abstract

**Background:**

Non-tubercular mycobacteria (NTM) has not been given due attention till the recent epidemic of HIV. This has led to increasing interest of health care workers in their biology, epidemiology and drug resistance. However, timely detection and drug susceptibility profiling of NTM isolates are always difficult in resource poor settings like India. Furthermore, no standardized methodology or guidelines are available to reproduce the results with clinical concordance.

**Objective:**

To find an alternative and rapid method for performing the drug susceptibility assay in a resource limited settings like India, we intended to evaluate the utility of Tetrazolium microplate assay (TEMA) in comparison with proportion method for reporting the drug resistance in clinical isolates of NTM.

**Methods:**

A total of fifty-five NTM isolates were tested for susceptibility against Streptomycin, Rifampicin, Ethambutol, Ciprofloxacin, Ofloxacin, Azithromycin, and Clarithromycin by TEMA and the results were compared with agar proportion method (APM).

**Results:**

Of the 55 isolates, 23 (41.8%) were sensitive to all the drugs and the remaining 32 (58.2%) were resistant to at least one drug. TEMA had very good concordance with APM except with minor discrepancies. Susceptibility results were obtained in the median of 5 to 9 days by TEMA. The NTM isolates were highly sensitive against Ofloxacin (98.18% sensitive) and Ciprofloxacin (90.09% sensitive). *M. mucogenicum *was sensitive only to Clarithromycin and resistant to all the other drugs tested. The concordance between TEMA and APM ranged between 96.4 – 100%.

**Conclusion:**

Tetrazolium Microplate Assay is a rapid and highly reproducible method. However, it must be performed only in tertiary level Mycobacteriology laboratories with proper bio-safety conditions.

## Background

The number and species of Non-tubercular mycobacteria (NTM) isolated from clinical specimens is continuously increasing with the advancement of microbiological detection methods. Numbers of reports have confirmed the pathogenic role, morbidity and mortality caused by NTMs in AIDS patients [1-4]. The infection due to NTM is difficult to treat because of their intrinsic resistance to the major classes of drugs, probably due to their habitat [5,6]. Therefore to choose an effective therapeutic drug, anti mycobacterial susceptibility testing becomes a primary step for the management of the NTM disease. Though there are specific recommendations and guidelines prescribed by the World Health Organization (WHO) and Centre for Disease Control (CDC) regarding anti-tubercular drug susceptibility methods for *Mycobacterium tuberculosis *there are no such guidelines for NTMs. As routine drug susceptibility testing of NTMs are discouraged, there are circumstances where susceptibility testing is warranted [7].

Traditionally drug susceptibility testing in mycobacterial isolates is performed in agar and Lowenstein Jensen (LJ) medium which is considered as a 'gold standard' but it is cumbersome and available only in few reference laboratories in a country like India where tuberculosis is highly endemic. Even though automated systems dramatically reduced the time for detecting drug resistance in mycobacterial isolates but these are more expensive and not feasible especially in a low resource setting [8-12].

Since there was a need for rapid, easy to perform and qualitative method for predicting the Minimal Inhibitory Concentration (MICs) for NTM isolates, Mshana et al [13] developed a rapid colorimetric method employing oxidation-reduction indicator Tetrazolium bromide to perform drug susceptibility testing for *Mycobacterium tuberculosis *which was found to be promising and cost effective [14].

Therefore, we intended to assess the performance and efficiency of Tetrazolium Microplate Assay (TEMA) for determining MICs of Streptomycin (STR), Rifampicin (RIF), Ethambutol (ETH), Ciprofloxacin (CIP), Ofloxacin (OFL), Azithromycin (ATH) and Clarithromycin (CLA) against clinically isolated NTMs. The results obtained by TEMA were compared with Agar Proportion Method (APM) on Middle brook 7H10 agar plates.

## Methods

### Mycobacterial isolates

A total of fifty-five NTMs isolated from patients referred to our laboratory for mycobacterial isolation were included, as shown in Table 1. These strains were identified and confirmed as NTM by biochemical methods such as heat stable catalase, niacin and nitrate production, aryl sulphatase and sodium chloride tolerance test [15] and DNA sequencing was done with targets of 16S rRNA and 16S-23S Internal Transcribed Spacer sequences or *hsp65 *as previously reported elsewhere [16]. Standard strains [*M. avium *(NCTC-8551), *M. chelonae *(TMC-1544), *M. xenopi *(NCTC-10042), *M. phlei *(NCTC-8156), *M. intracellularae *(TMC1406), *M. simiae *(TMC 1226), *M. fortuitum *(TMC1529), *M. smegmatis *(TMC1546), *M. terrae *(TMC-1450) &* M. kansasii*] used as controls against the clinical NTM isolates were kindly gifted by Dr. V. M. Katoch, National JALMA Institute of Leprosy and Other Mycobacterial Diseases, Agra, India.

**Table 1:** Mean MICs of drugs (μg/mL) against clinical isolates of Non-tubercular mycobacterial isolates

**Clinical isolate (n)**	**STR**		**RIF**		**ETH**		**CIP**		**OFL**		**ATH**		**CLA**	
							
	**M**	**SD**	**M**	**SD**	**M**	**SD**	**M**	**SD**	**M**	**SD**	**M**	**SD**	**M**	**SD**
*M. avium *(16)	0.6	1.69	0.5	0.96	57.7	66.63	0.27	0.48	0.6	0.16	158.5	215.9	22.2	32.53
*M. abscess *(1)	0.22	-	0.22	-	138.3	-	0.13	-	0.66	-	1.37	-	0.16	-
*M. chelonae *(7)	3.98	10.49	1.03	2.58	19.98	52.17	0.13	-	0.66	-	1.95	1.55	0.23	0.18
*M. fortuitum *(7)	0.05	-	0.29	0.63	19.98	52.17	0.13	-	0.66	-	36.8	93.97	12.02	31.38
*M. intracellulare *(1)	0.54	-	0.54	-	0.27	-	0.13	-	0.66	-	1.37	-	0.16	-
*M. kansasii *(1)	0.21	-	0.05	-	0.27	-	0.13	-	0.66	-	1.37	-	0.32	-
*M. mucogenicum *(1)	0.86	-	27.6	-	138.3	-	0.53	-	2.06	-	43.75	-	1.3	-
*M. parascrofulaceum *(1)	1.72	-	0.05	-	138.3	-	0.13	-	0.66	-	1.37	-	0.16	-
*M. phlei *(3)	0.12	0.08	0.12	0.08	49.07	77.38	0.77	1.11	0.66	-	59.24	100.24	27.84	47.94
*M. scrofulaceum *(1)	0.21	-	0.05	-	0.27	-	0.13	-	0.66	-	1.37	-	0.32	-
*M. simiae *(4)	0.47	0.83	0.92	1.68	69.28	79.69	4.26	8.16	0.66	-	1.37	-	0.16	-
*M. smegmatis *(3)	0.11	0.093	0.54	-	46.28	79.69	0.13	-	0.66	-	30.07	49.72	7.04	11.91
*M. terrae *(7)	1.81	2.57	1.87	2.54	20.37	52.01	0.26	0.33	0.6	0.14	1.37	-	12.02	31.38
*M. xenopi *(2)	0.05	-	0.05	-	0.27	-	0.13	-	0.66	-	1.37	-	0.16	-

STR: Streptomycin, RIF: Rifampicin, ETH: Ethambutol, CIP: Ciprofloxacin, OFL: Ofloxacin, ATH: Azithromycin, CLA: Clarithromycin, M: Mean, SD: Standard deviation.

### Inoculum and drug preparation

Antimycobacterial drugs were procured from commercial source (Sigma^®^, U.S.A) and solubilised according to the manufacturer's recommendations in appropriate solvents and sterilized by filtering through 0.22 μm membrane filter (Millipore^®^, Ireland). Tetrazolium bromide [3-(4, 5-dimethylthiazol-2-yl)-2,5-diphenyl-tetrazolium bromide] (Sigma^®^, USA.) was prepared at a concentration of 1 mg/ml in absolute ethanol and 1.5 ml of 10% Tween 80 and the stock was stored in dark at 4°C. The mycobacterial inoculum was prepared from log-phase culture of the NTMs on LJ slants and their turbidity was adjusted to McFarland Standard No. 1.

### Tetrazolium Microplate Assay

**Figure 1:**
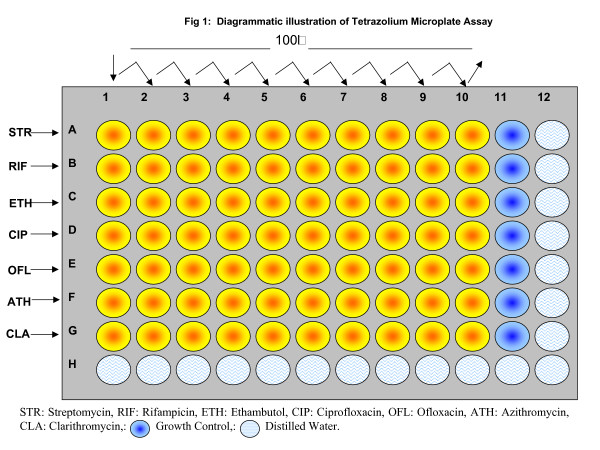
Diagrammatic illustration of tetrazolium microplate assay.

TEMA was performed as previously described [13] with minor modifications (Illustrated in Fig. 1). Briefly, 100 μL of Middlebrook 7H9 broth (pH 7.2; Sigma^®^, USA) was added to columns 2 to 11 in rows A to G (labeled on microtitre plates). One-hundred-microliters of 2× concentration of drug were added to columns 1 and 2. The antibiotics were serially diluted twofold in consecutive columns by transferring 100 μL, except for column 10, where 100 μL of excess medium was discarded. The final drug concentrations in the wells were set as follows: STR: 27.6 to 0.0539 μg/mL, RIF: 27.6 to 0.0539 μg/mL, ETH: 138.3 to 0.27 μg/mL, CIP: 66 to 0.132 μg/mL, OFL: 33 to 0.66 μg/mL, ATH: 500 to 1.367 μg/mL and CLA 83.2 to 0.162 μg/mL. Hundred microliters of mycobacterial suspension (set to McFarland Standard No.1) was added to wells in rows A to G in columns 1 to 11. The wells in column 11 served as inoculum-growth control with no drugs. The plates were incubated at 37°C for 5 days. On day 5, 50 μl of the tetrazolium dye was added to well A11 and the plate was then incubated at 37°C for 24 h. A change in colour from yellow to purple indicated growth of bacteria and the MICs was interpreted visually.

### Agar Proportion method

APM method was performed on Middlebrook 7H10 agar plates as previously described by others [15,17]. The drug concentrations in the agar plates were STR 8 μg/mL, RIF 2 μg/mL, ETH 40 μg/mL, CIP 2 μg/mL, OFL 2 μg/mL, ATH 200 μg/mL, and CLA 80 μg/mL. The strains were classified as susceptible to a drug if the number of colonies was <1% and resistant if the number of colonies was >10% in the drug containing media to the number of colonies on the control plate without any drug.

### Data analysis

Analysis of data obtained by TEMA and the APM was carried by SPSS^® ^software (version 11.5). Receiver Operating Characteristic (ROC) curve analysis was performed to measure the Area Under Curve (AUC).

## Results

**Figure 2:**
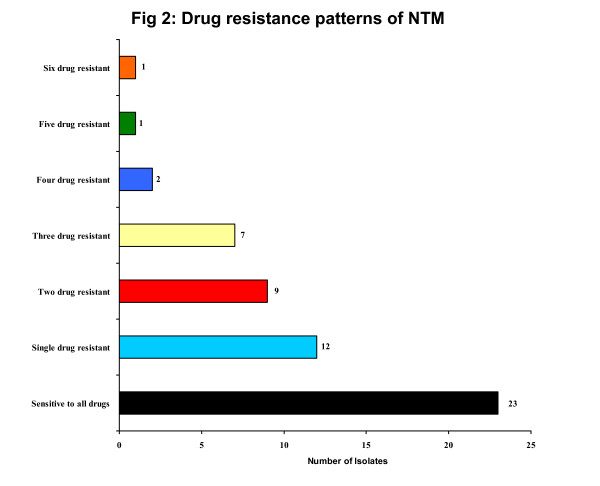
Drug resistance patterns of NTM.

The sensitivity and resistance patterns against STR, RIF, ETH, CIP, OFL, ATH and CLA by both TEMA and APM are described in Table 1. All the standard strains of NTMs were sensitive to all the tested drugs. The mean MICs values of the drugs (Table 2) tested by TEMA varied between clinical isolates belonging to different species. Of the 55 isolates, 23 (41.8%) were sensitive to all the drugs and the remaining 32 (58.2%) were resistant to at least one drug (Fig. 2).

**Table 2:** Comparison of TEMA and APM for antimycobacterial susceptibility testing of Non-tubercular mycobacterial isolates

	**APM**													
	
	**STR**		**RIF**		**ETH**		**CIP**		**OFL**		**ATH**		**CLA**	
							
**TEMA**	**S**	**R**	**S**	**R**	**S**	**R**	**S**	**R**	**S**	**R**	**S**	**R**	**S**	**R**
	
**Sensitive**	43	1	45	0	36	0	50	0	53	0	43	0	45	0
**Resistant**	1	10	1	9	0	19	0	5	1	1	1	11	1	9
**Total**	44	11	46	9	36	19	50	5	54	1	44	11	46	9
**Percentage**	(80%)	(20%)	(83.6%)	(16.4%)	(65.5%)	(34.5%)	(91%)	(9%)	(98.2%)	(1.8%)	(80%)	(20%)	(83.6%)	(16.4%)
*Concordance*	96.4%		98%		100%		100%		98%		98%		98%	

TEMA: Tetrazolium Microplate Assay, STR: Streptomycin, RIF: Rifampicin, ETH: Ethambutol, CIP: Ciprofloxacin, OFL: Ofloxacin, ATH: Azithromycin, CLA: Clarithromycin, S: Sensitive, R: Resistant.

**Figure 3:**
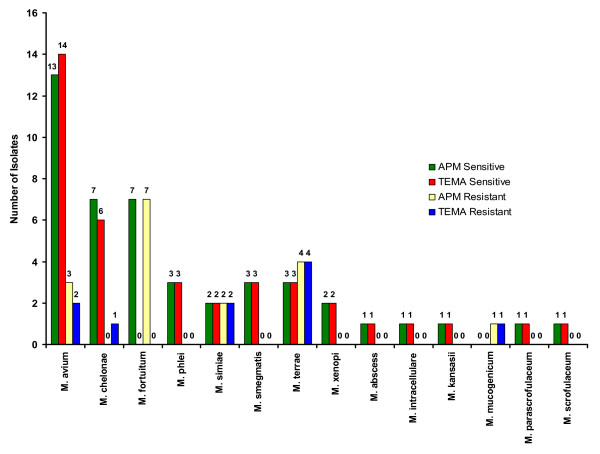
Drug resistance patterns in individual species of NTM against Streptomycin.

**Figure 4:**
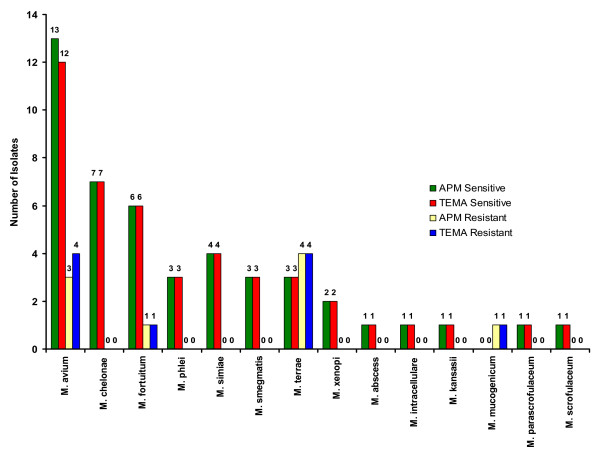
Drug resistance patterns in individual species of NTM against Rifampicin.

**Figure 5:**
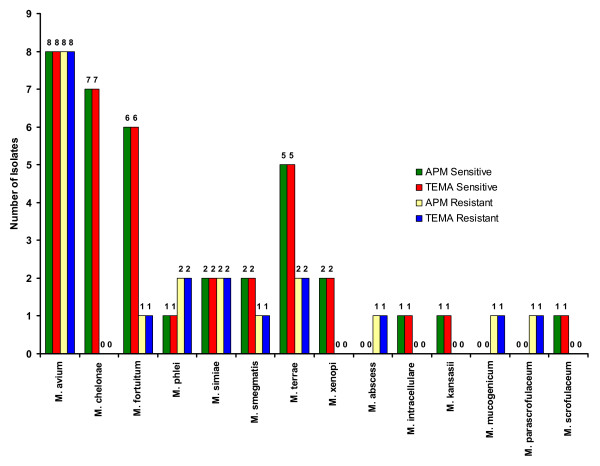
Drug resistance patterns in individual species of NTM against Ethambutol.

**Figure 6:**
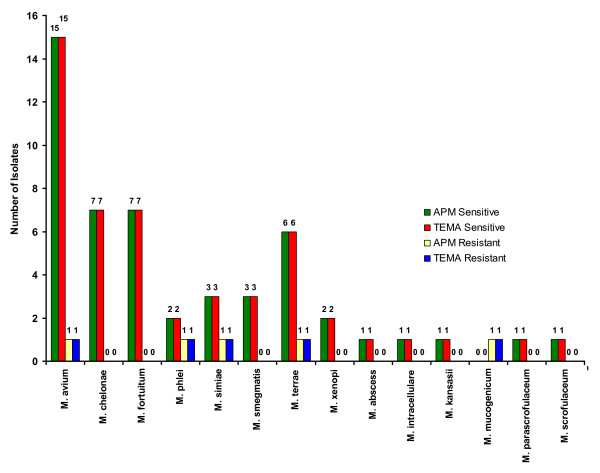
Drug resistance patterns in individual species of NTM against Ciprofloxacin.

**Figure 7:**
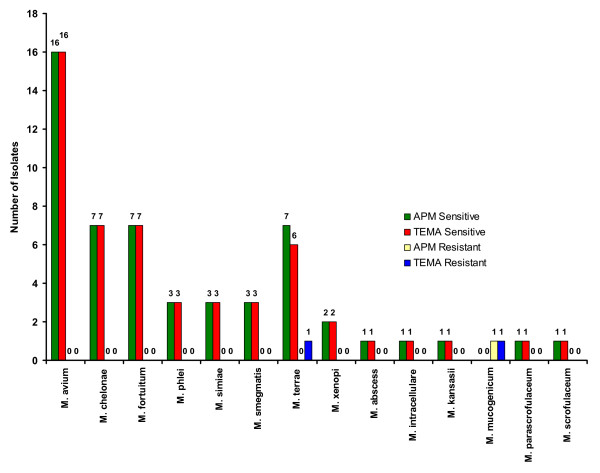
Drug resistance patterns in individual species of NTM against Ofloxacin.

**Figure 8:**
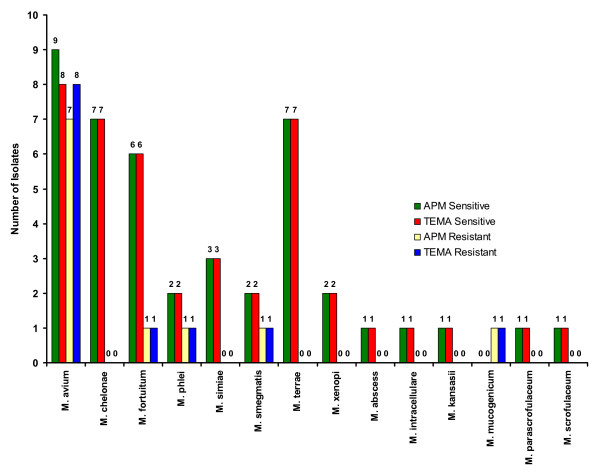
Drug resistance patterns in individual species of NTM against Azithromycin.

**Figure 9:**
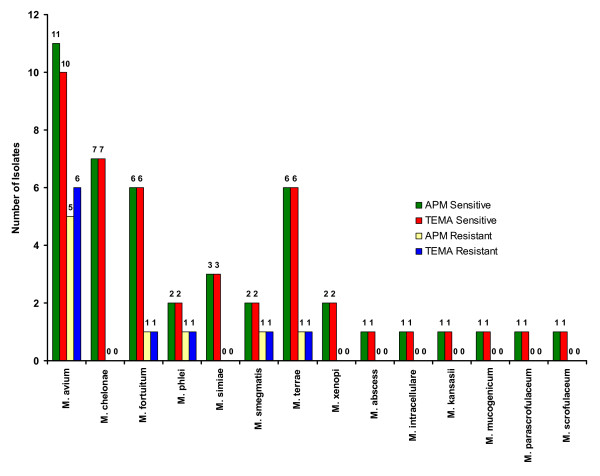
Drug resistance patterns in individual species of NTM against Clarithromycin.

When the efficiency of TEMA and APM were compared for detecting susceptibility patterns in NTM isolates, they showed 96.4% concordance for STR (Fig. 3), 98% concordance for RIF (Fig. 4), 100% for ETH (Fig. 5), and CIP (Fig. 6), and 98% for OFL (Fig. 7), ATH (Fig. 8) and CLA (Fig. 9). The Area Under Curve (AUC) by ROC curve analysis for STR was 0.943, RIF was 0.950, ETH was 1.000, CIP was 1.000, OFL was 0.750, ATH was 0.958 and CLA was 0.950. NTM isolates showed 90.09% and 98.18% of susceptibility to Ciprofloxacin and Ofloxacin, respectively. TEMA was rapid and susceptibility results could be obtained in a median time period of 5 to 9 days as compared to 12 to 25 days by APM.

## Discussion

The epidemiology of mycobacterial infections had changed drastically in the past few decades and in-turn changing the treatment regimen and management practices, especially after AIDS epidemic [18]. In the terminal stages of AIDS, where CD_4_^+ ^lymphocyte counts falls below 100, not only *M. tuberculosis *but several NTM also cause infection and can be isolated from blood, tissue, sputum and fecal samples [19]. The NTM disease not necessarily remains localized, but cause disseminated infection such as *M. avium-intracellulare *complex (MAC) [20]. The prognosis for the MAC infected AIDS patient is usually poor, largely due to the inherent resistance of these organisms to most of the available anti-tubercular drugs and have high toxicity.

Seventeen (30.9%) of the 55 NTM isolates studied here were isolated from patients who were earlier treated with anti-tuberculosis therapy (ATT) without any clinical improvement. In clinical settings, these non-responsive cases are later labeled as Multi-drug resistant tuberculosis (MDR) by missing NTM infections. Similar observations were reported with other studies carried out in other countries such as Iran [21] and Brazil [22]. The clinical and radiological features of NTM infected patients like abnormal chest roentgenograms with infiltrations, nodular abscesses, cavities, lymphoadenopathy resembling TB can mislead the physician.

TEMA was effectively used to detect drug resistance in NTM isolates and was compared with the APM to which it had very good concordance (table 2). Previously similar results were obtained with *M. tuberculosis *(Data not published), prompted us to evaluate with other mycobacterial species. In this regard, the TEMA results were accurate, highly reproducible and rapid to determine the MICs of clinically significant mycobacteria.

However, before applying the TEMA as a routine drug susceptibility testing method, a multicentric and inter laboratory testing must be carried out with a supervision of international agencies such as World Health Organisation to validate our findings and to implement it in resource poor settings. We recommend that the TEMA, which is a calorimetric method, is more appropriate for level 3 reference laboratories to manipulate small volume of liquid cultures in a microplate format.

## Competing interests

The authors declare that they have no competing interests.

## Authors' contributions

MMS participated in the collection and revival of strains, susceptibility testing, drafted the manuscript and performed the statistical analysis. KG participated in the design of the study, collection and characterization of strains. RS provided the clinical samples. SS conceptualized and designed the study. He also coordinated the study, critically evaluated the paper and arranged financial support. All authors have read and approved the final manuscript.
